# Proteomics study the potential targets for Rifampicin-resistant spinal tuberculosis

**DOI:** 10.3389/fphar.2024.1370444

**Published:** 2024-04-15

**Authors:** Yanling Wang, Shijie Yin, Shixiong Wang, Kuan Rong, Xiang-He Meng, Huashan Zhou, Luo Jiao, Da Hou, Zhongjing Jiang, Jun He, Zenghui Mao

**Affiliations:** ^1^ Hunan Provincial Key Laboratory of Regional Hereditary Birth Defects Prevention and Control, Changsha Hospital for Maternal & Child Healthcare Affiliated to Hunan Normal University, Changsha, China; ^2^ College of Life Science, Hunan Normal University, Changsha, China; ^3^ Hunan Academy of Traditional Chinese Medicine Affiliated Hospital, Changsha, China; ^4^ Department of Spine Surgery and Orthopaedics, Xiangya Hospital, Central South University, Changsha, China

**Keywords:** DSTB, NDSTB, HLA, DEPs, MMP9, PLCL1

## Abstract

**Introduction:** The escalating global surge in Rifampicin-resistant strains poses a formidable challenge to the worldwide campaign against tuberculosis (TB), particularly in developing countries. The frequent reports of suboptimal treatment outcomes, complications, and the absence of definitive treatment guidelines for Rifampicin-resistant spinal TB (DSTB) contribute significantly to the obstacles in its effective management. Consequently, there is an urgent need for innovative and efficacious drugs to address Rifampicin-resistant spinal tuberculosis, minimizing the duration of therapy sessions. This study aims to investigate potential targets for DSTB through comprehensive proteomic and pharmaco-transcriptomic analyses.

**Methods:** Mass spectrometry-based proteomics analysis was employed to validate potential DSTB-related targets. PPI analysis confirmed by Immunohistochemistry (IHC) and Western blot analysis.

**Results:** The proteomics analysis revealed 373 differentially expressed proteins (DEPs), with 137 upregulated and 236 downregulated proteins. Subsequent Gene Ontology (GO) and Kyoto Encyclopedia of Genes and Genomes (KEGG) analyses delved into the DSTB-related pathways associated with these DEPs. In the context of network pharmacology analysis, five key targets—human leukocyte antigen A chain (HLAA), human leukocyte antigen C chain (HLA-C), HLA Class II Histocompatibility Antigen, DRB1 Beta Chain (HLA-DRB1), metalloproteinase 9 (MMP9), and Phospholipase C-like 1 (PLCL1)—were identified as pivotal players in pathways such as “Antigen processing and presentation” and “Phagosome,” which are crucially enriched in DSTB. Moreover, pharmaco-transcriptomic analysis can confirm that 58 drug compounds can regulate the expression of the key targets.

**Discussion:** This research confirms the presence of protein alterations during the Rifampicin-resistant process in DSTB patients, offering novel insights into the molecular mechanisms underpinning DSTB. The findings suggest a promising avenue for the development of targeted drugs to enhance the management of Rifampicin-resistant spinal tuberculosis.

## Introduction

Tuberculosis (TB) is a communicable disease resulting from *Mycobacterium tuberculosis* (*M. tuberculosis*) infection (Nahid et al., 2016; Sable et al., 2019). The global impact of TB in 2021 was staggering, with an estimated 10.6 million people falling ill, equating to 134 cases per 100,000 population. Geographically, South-East Asia accounted for 45% of TB cases, underscoring its pervasive presence (Bagcchi, 2023). Within the spectrum of TB cases, 10% manifest as extrapulmonary TB, with half affecting the musculoskeletal system. The spine, comprising 1%–2% of cases, emerges as the most prevalent site in extrapulmonary TB (Dunn and Ben Husien, 2018).

However, Rifampicin resistance in *M. tuberculosis*, the causative agent of tuberculosis (TB), is a growing global concern that has significant implications for public health. Studies have indicated that over 90% of tuberculosis cases resistant to Rifampicin also exhibit resistance to isoniazid, establishing Rifampicin resistance as a reliable surrogate indicator for multidrug-resistant TB (MDR-TB) (Morgan et al., 2005; Cavusoglu et al., 2002).The emergence of drug-resistant strains of *M. tuberculosis*, particularly MDR-TB and extensively drug-resistant TB (XDR-TB), presents a formidable challenge to TB control efforts (Bagcchi, 2023). The proliferation of drug-resistant TB strains not only complicates treatment but also heightens mortality rates, amplifies healthcare costs, and intensifies the risk of further transmission (Zumla et al., 2015). Thus, this sparked interest in exploring the mechanism of Rifampicin-resistant for new multidrug-resistant therapy.

The crux of drug resistance in clinical *M. tuberculosis* (MTB) strains predominantly resides in chromosomal mutations, precipitating several pivotal mechanisms. These mechanisms include alterations in drug targets through genetic changes, reducing susceptibility to anti-TB drugs. Overexpression of drug targets further diminishes drug effectiveness, while disruptions in prodrug activation impede the conversion of prodrugs into active forms within bacterial cells (Lata et al., 2015; de Keijzer et al., 2016; Banaei-Esfahani et al., 2017). Additionally, the activation of efflux pumps, specialized transport proteins expelling drugs from bacterial cells, contributes to resistance against anti-TB drugs (Miotto et al., 2018). However, the challenge of diminishing drug resistance or discovering novel and effective treatments for Rifampicin-resistant spinal tuberculosis (TB) or extensively drug-resistant TB (XDR-TB) persists without a clear solution.

**FIGURE 1:**
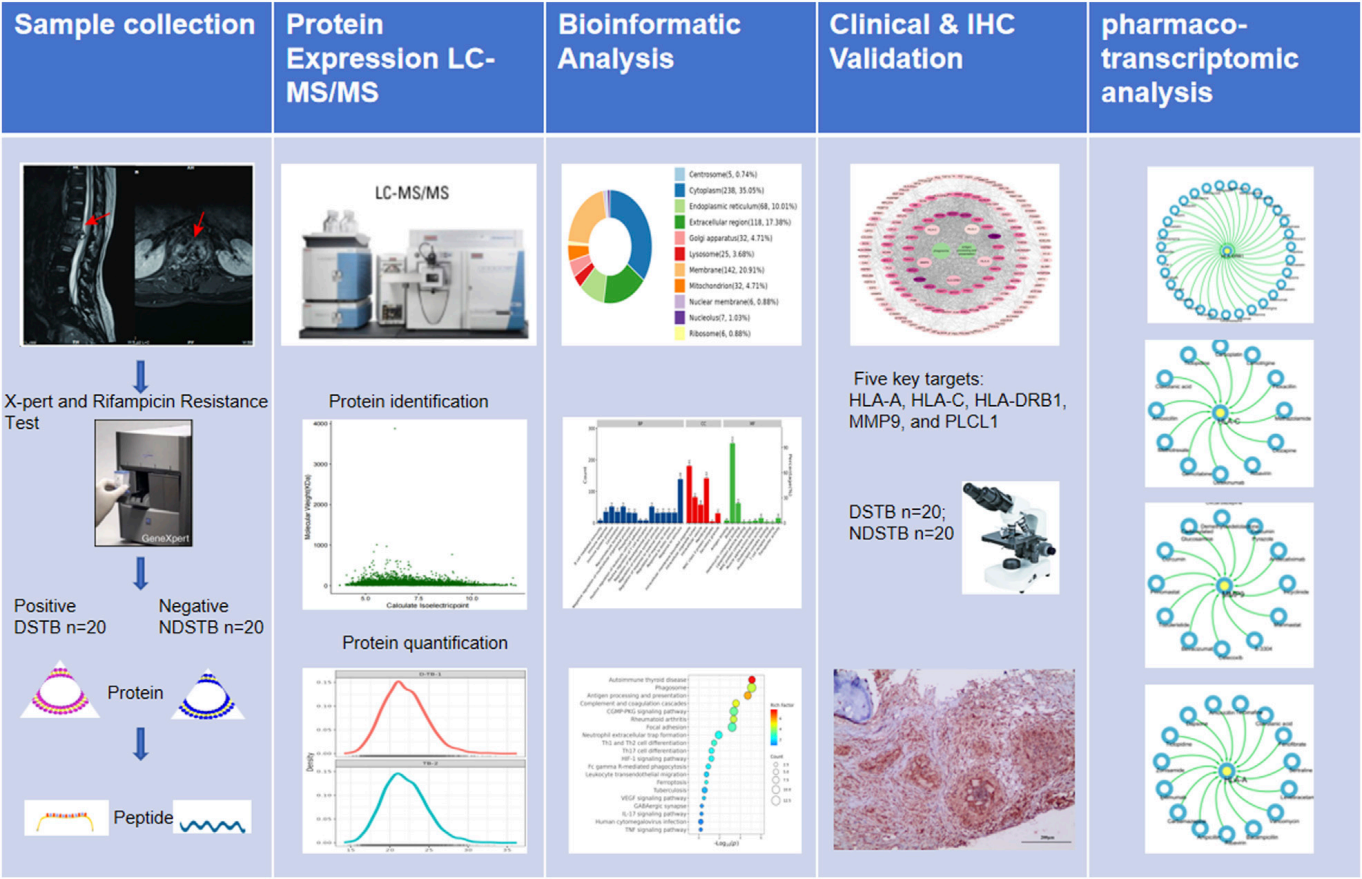
The graphical abstract of our study was presented.

In this study, our primary focus was to identify potential targets based on proteomics and explore the mechanism of Rifampicin-resistance using pharmaco-transcriptomic analyses. The graphical abstract of our study was presented in Figure 1.

## Materials and methods

### Clinical sample acquisition

We have secured the proper approval from the Ethics Committee of Hunan Academy of Traditional Chinese Medicine Affiliated Hospital for our study. The investigation involved the examination of forty cases of spinal tuberculosis lesion tissues, collected from the Hunan Academy of Traditional Chinese Medicine Affiliated Hospital. This dataset encompassed 20 patients with non Rifampicin-resistant spinal tuberculosis (NDSTB) forming the control group, and an additional 20 patients with Rifampicin-resistant spinal tuberculosis (DSTB) constituting the experimental group. Surgical removal of spinal tuberculosis lesions was performed on all patients between January 2020 and June 2023, adhering to the inclusion and exclusion criteria outlined in Table 1. Clinical information, including sex, age, history of tuberculosis, regular use of anti-tuberculosis drugs, and T-SPOT.TB test results (Table 2), and the site of spinal infection were meticulously extracted from medical records. After the surgeries, postoperative pathological examinations and culture results unequivocally confirmed the presence of MTB infection in the collected tissue samples. This robust confirmation served as the cornerstone for the study’s investigation into Rifampicin-resistance in spinal tuberculosis.

**TABLE 1:** Inclusion and exclusion criteria of DSTB and NDSTB.

NDSTB
Inclusion Criteria
(1) Initially, study participants were diagnosed with spinal tuberculosis (STB) based on clinical symptoms and test results by two or more specialists. In the first step, both clinical Xpert Resistance test and Rifampicin Resistance (RIF) results were negative for all participants
(2) Age≥18
(3) Infections of tuberculous origin characterized by low fevers, night sweats, weight loss, and fatigue
(4) All participants were treated with four (isoniazid, rifampicin, pyrazinamide (PZA) and ethambutol (EMB) (category I treatment)) drugs every day for 2 weeks before debridement to treat Spinal Tuberculosis (STB)
(6) The participants’ Informed consent had been obtained
**Exclusion criteria**
(1) Comorbidity with diseases affecting STB: such as immunodeficiency, etc.
(2) The participants were not treated with four (isoniazid, rifampicin, pyrazinamide (PZA) and ethambutol (EMB) (category I treatment)) drugs or treated with other medicine
(3) Study participants with STB who did not provide informed consent
**DSTB**
**Inclusion Criteria**
(1) Initially, study participants were diagnosed with spinal tuberculosis (STB) based on clinical symptoms and test results by two or more specialists. In the first step, both clinical Xpert Resistance test and Rifampicin Resistance (RIF) results were positive for all participants
(2) Age≥18
(3) Infections of tuberculous origin characterized by low fevers, night sweats, weight loss, and fatigue
(4) All participants were treated with four (isoniazid, rifampicin, pyrazinamide (PZA) and ethambutol (EMB) (category I treatment)) drugs every day for 2 weeks before debridement to treat Spinal Tuberculosis (STB)
(5) The participants’ Informed consent had been obtained
**Exclusion criteria**
(1) Comorbidity with diseases affecting STB: such as immunodeficiency, etc.
(2) The participants were not treated with four (isoniazid, rifampicin, pyrazinamide (PZA) and ethambutol (EMB) (category I treatment)) drugs or treated with other medicine
(3) Study participants with STB who did not provide informed consent

**TABLE 2:** Baseline characteristics of participants and comparison between NDSTB groups and DSTB group. Participants without a tuberculosis history or a regular use of anti-tuberculosis drugs were not documented. Additionally, any test results that went undetected were not recorded.

	All	NDSTB	DSTB
Subjects, n	40 (100%)	20 (50.0%)	20 (50.0%)
Age, y	53.3 ± 14.2	55.6 ± 12.3	51.1 ± 15.7
Sex			
Male	25 (62.5%)	14 (%)	11 (%)
Female	15 (37.5%)	6 (%)	9 (%)
History of tuberculosis	5 (100%)	3 (60%)	2 (40%)
History of regular use of anti-tuberculosis drugs	4 (100%)	2 (50%)	2 (50%)
diagnosed as spinal tuberculosis	40 (100%)	20 (50.0%)	20 (50.0%)
T-SPOT.TB test results (positive)	35 (87.5%)	18 (51.4%)	17 (49.6%)
Bacterial culture results (positive)	11 (100%)	5 (45%)	6 (55%)
Bacterial culture results (drug-resistant)	11 (100%)	5 (45%) negative	6 (55%) positive
X-pert Resistance Test	40 (100%)	20 (50%) negative	20 (50%) positive
Rifampicin Resistance Test	40 (100%)	20 (50%) negative	20 (50%) positive

### Protein extraction

First, we obtained tissues we obtained within 30 min of surgical removal and the samples were rapidly frozen using liquid nitrogen. Then, the frozen tissue samples were preserved in a −80°C refrigerator for subsequent analysis. The tissues of spinal tuberculosis lesions were thawed on ice and then lysed with a lysis buffer (Beyotime, P0013J, Shanghai, China). Subsequently, we added 1% PMSF (Beyotime, ST507-10 mL) and 1% inhibitor cocktail (Roche, 4693116001, Basle, Switzerland) and lysed the mixture for 40 min. Further, the lysis samples were centrifuged for 25 min at 4°C, 12,000 g in a 1.5 mL EP tube. The supernatant was isolated and quantified protein concentration in a new tube.

### Sample preparation

The process of ultrafiltration (Microcon units, 30 kD) was repeated several times using 200 µL of UA buffer (8 M Urea, 150 mM Tris-HCl pH 8.0) in combination with centrifugation to remove the detergent and other low-molecular-weight components from the mixture. To block reduced cysteine residues, we added 100 µL of 0.05 M iodoacetamide in the UA buffer. Subsequently, the lucifugous precipitate was incubated for 20 min. To proceed, we had to properly wash the sample, so 25 mM NH4HCO3 was added twice for 100 µL. Next, Trypsin (Promega) was used to digest the protein suspension overnight at 37°C in 40 µL of 25 mM NH4HCO3. The filtrate peptides were concentrated with OD_280_ using a Nanodrop device.

### Tandem mass tags labeling and Desalting of peptides

The peptide mixtures underwent labeling with TMT reagents (Thermo Scientific, CA, United States) following the manufacturer’s instructions. Each aliquot (100 µg of peptide equivalent) was treated with one tube of TMT reagent. Subsequently, 100 µL of 0.05 M TEAB (pH 8.5) was added to the mixtures. The TMT reagents were dissolved in 41 µL of 100% acetonitrile (ACN) before being applied for labeling at room temperature. Following the addition of TMT reagents to the peptide mixtures, the combined solutions were incubated for 1 hour. To halt the reaction, 8 µL of 5% hydroxylamine was introduced and allowed to incubate for 15 min. The Multiplex labeled samples were then pooled together and subjected to lyophilization.

### High pH reverse phase fractionation and Nano LC-MS analysis

Sample labeled TMT fractionation was completed using Waters XBridge BEH130 column (C18, 3.5 μm, 2.1 × 150 mm) on an Agilent 1290 HPLC operating at 0.3 mL/min. We finished the elution of peptides using buffers (Buffer A: 10 mM ammonium formate; Buffer B: 10 mM ammonium formate with 90% ACN, PH 10) at a flow rate of 100 μL/min. For each peptide mixture, 30 fractions were collected, and they were concatenated into 15 final ones (pooling equal intervals of RPLC fractions). Elution materials were freeze-dried using vacuum centrifugation for LC–MS analysis (−20°C stored).

The lyophilized eluates underwent analysis using Easy nLC (Ultimate RSLC Nano, Thermo Scientific), equipped with a C18-reversed phase column (12 cm long, 75 μm ID, 3 μm). The sample was absorbed by buffer A (2% ACN and 0.1% Formic acid) and subsequently separated by buffer B (90% ACN and 0.1% Formic acid) at a flow rate of 300 nL/min for a duration of 90 min. The gradient used was as follows: 0–2 min, 2%–5% buffer B; 2–62 min, 5%–20% buffer B; 62–80 min, 20%–35% buffer B; 80–83 min, 35%–90% buffer B; 83–90 min, buffer B 90%. Data of MS was collected by dynamically selected the most abundant precursor ions from the survey scan (300–1800 m/z). This was followed by a full Orbitrap MS scan (AGC target: 1e6, maximum injection time: 50 ms), and data-dependent Orbitrap MS/MS scans (isolation window = 1.5, injection time: 100 ms, activation type: HCD, AGC target: 1e5, max.) with a dynamic exclusion duration of 30 s. The resolution for survey scans was set at 70,000 at m/z 200, and for HCD spectra, it was set at 35,000 at m/z 200. The collision energy normalized to 30 was measured, and the instrument was configured with the peptide recognition mode enabled.

### Data analysis and bioinformatics analysis

Our data analysis was demonstrated with Proteome Discoverer Version 1.6.0.16 and an initial mass windows of 10 ppm were used as precursor. We properly followed the cleavage rules of Trypsin/P, so a maximum of two missed cleavage sites were allowed, as well as a mass tolerance of 20 ppm for fragment ions. Fixed modification: Carbamidomethyl (C), TMT10plex(K), TMT10plex (N-term), Variable modification: Oxidation(M), and Acetyl (Protein N-term).

Differentially significant expressed proteins (DEP) were screened using log2FC > 1 or log2FC ≤ −1; *p* < 0.05 as the threshold and protein-level hierarchical clustering was used to group expression data. We annotated the sequences using UniProtKB/Swiss-Prot, KEGG, and Gene Ontology (GO) and also conducted Fisher’s exact test to analyze GO and KEGG enrichment. All genes were ranked based on their degree of differential expression in the two groups of samples, followed by applying a statistical method to assess whether the predetermined set of genes was enriched at the top or bottom of the ranked list. Gene Set Enrichment Analysis (GSEA) involves three primary steps: calculating the Enrichment Score, estimating the significance level of the Enrichment Score, and conducting multiple hypotheses testing. GSEA utilizes both Gene Ontology (GO) and Kyoto Encyclopedia of Genes and Genomes (KEGG) databases. In the results of GO analysis, gene sets represent GO terms, while in KEGG analysis, gene sets represent individual pathways.

### Construction of a PPI network of DEPs proteins in Rifampicin-resistant spinal TB and “antigen processing and presentation” and “Phagosome” pathways

Key targets in Rifampicin-resistant spinal tuberculosis were identified by intersecting two disease-related pathways in WGCNA with DEPs (version 7.5.1). The obtained results were utilized to construct a protein-protein interaction network using the STRING database and visualized through Cytoscape (version 3.9.0).

### Pharmaco-transcriptomic analysis

To propose novel treatment strategies for Rifampicin-resistant spinal tuberculosis (TB), we performed a pharmaco-transcriptomic analysis using the Integration of the Drug–Gene Interaction Database (DGIdb 4.0). The DGIdb database (version 4.0) integrates information on drug-gene interactions and druggable genes sourced from publications, databases, and other web-based resources (Freshour et al., 2021). It serves as a comprehensive, publicly accessible web database. Finally, we utilized Cytoscape to assess and visualize the impact of drug molecule metabolism on the regulation of genes.

### Immunohistochemistry

Focal tissues from all patients in each group were immersed in 4% paraformaldehyde and subsequently embedded in paraffin. The paraffin-embedded slices underwent a 30-min incubation at 100°C. Following this, the samples were sequentially treated with 100% xylene, 95% xylene, 95% ethanol, 95% ethanol, and 100% ethanol, each for 5 min. Antigen repair of the slices was performed using citrate buffer. The tissues were then exposed to blocking buffer (20% heat-inactivated sheep serum, SP KIT-B2) at room temperature for 1 hour. Subsequently, they were incubated with primary antibodies (HLA-DRB1 (proteintech, 15862-1-AP), HLA-A (proteintech, 55383-1-AP), HLA-C (proteintech, 15777-1-AP), MMP9 (proteintech, 10375-2-AP), PLCL1 (Abcam, ab190225)) at a 1:50 dilution. Following a PBS wash for 5 min four times, the secondary antibody was applied for 1 hour. Once developed with DAB chromogen and counterstained with hematoxylin, the samples were observed under a microscope.

### Western blot analysis

The protein samples were loaded into wells containing an SDS-PAGE gel, and electrophoresis was carried out. A solution of TBS was used to prepare a 5% skimmed milk powder and 5% BSA protein blocking solution. After successful membrane transfer, the PVDF membrane was labeled and then incubated with the blocking solution. The blocking process was performed under gentle shaking at room temperature for 1–2 h. Subsequently, the PVDF membrane was incubated overnight at 4°C with the primary antibody solution (diluted with the appropriate diluent from Biyuntian Company, PLCL1 (Abcam, ab157200) at 1:1000, other primary antibodies at 1:1000). Following incubation, the PVDF membrane was washed with TBST solution at room temperature and then incubated with the secondary antibody. An appropriate amount of ECL luminous solution was added, and the target bands were detected using a chemiluminescence imaging system (CIIC). Images were captured, and the gray value of the protein bands was calculated using ImageJ software.

### Statistical analysis

The statistical analysis of our dataset was performed in SPSS 26 and GraphPad Prism 7. All the numerical data were expressed as mean ± SD. Comparisons between groups were conducted using ANOVA with Newman–Keuls *post hoc* tests and t-tests. Statistical significance was defined as *p* < 0.05.

## Results

### Participant baseline characteristics

**FIGURE 2:**
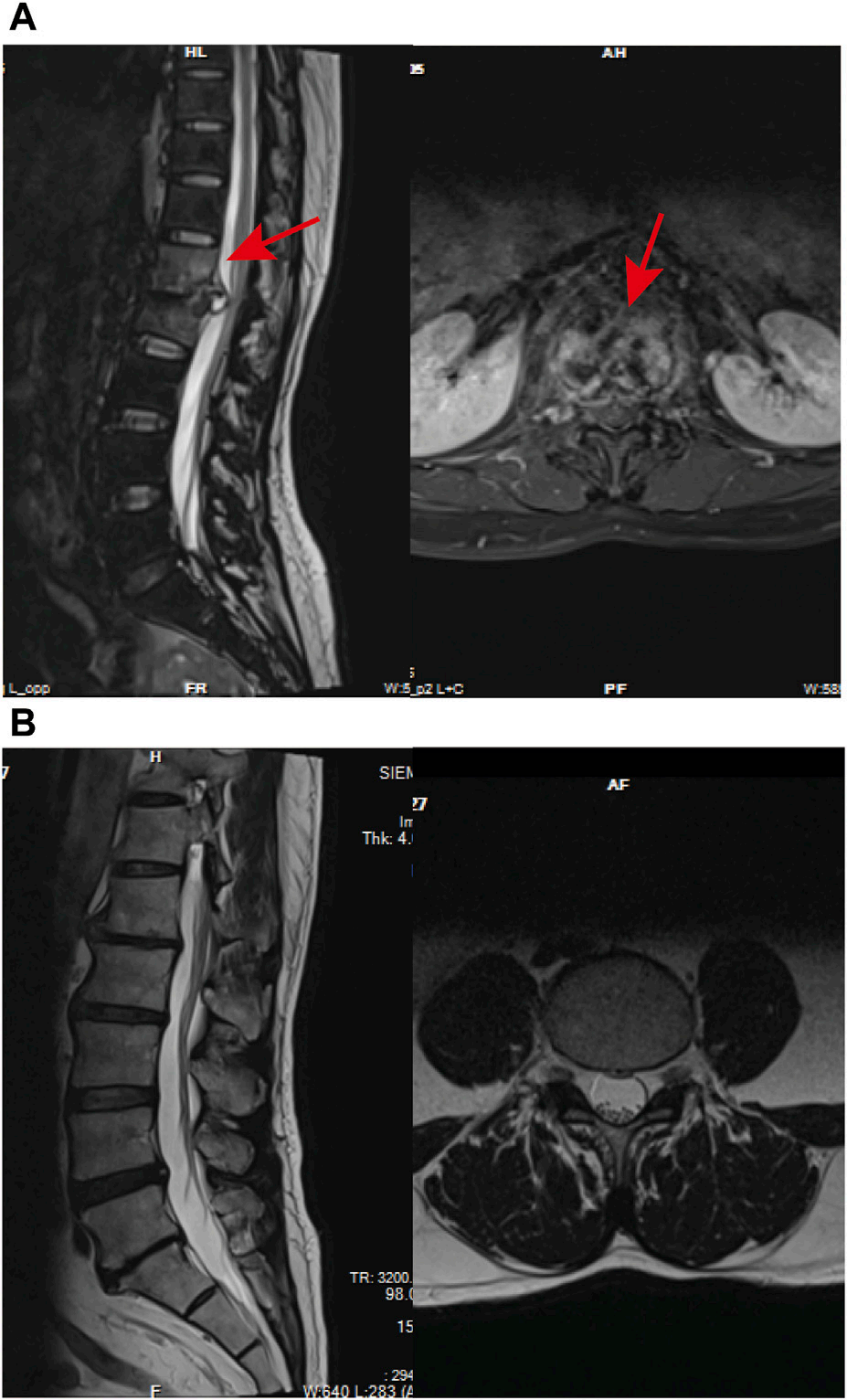
Radiographic manifestations of spinal tuberculosis. **(A)** Radiographic manifestations of spinal tuberculosis (indicated by arrows). **(B)** Imaging features of a normal spine.

In the NDSTB group, there were a total of 20 participants, including 6 females and 14 males, with an average age of 55.6 ± 12.3 years. The DSTB group consisted of 9 females and 11 males, with a mean age of 51.1 ± 15.7 years (Table 2). The results showed no significant difference in age and sex between the two groups (*p* = 0.117, *p* = 0.408). All participants underwent questioning and testing for radiographic manifestations, T-SPOT.TB, Bacterial culture, X-pert Resistance Test, and Rifampicin Resistance Test in hospitals, with statistics recorded accordingly to the actual situation. Additionally, radiographic manifestations of spinal tuberculosis were documented and compared with those of a normal spine (Figure 2).

### Protein identifcation

The protein expression profile in lesion locations from study participants with NDSTB and DSTB was investigated using TMT-based proteomics. This analysis aimed to assess the underlying mechanisms associated with Rifampicin-resistant spinal tuberculosis.

**FIGURE 3:**
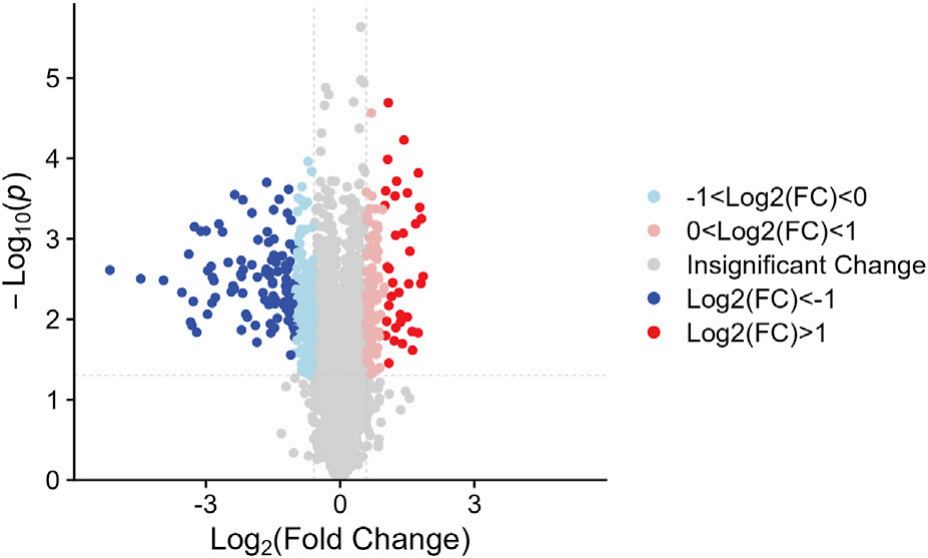
Two groups gomparison: Differential analysis showed that 373 were differentially expressed between the two groups with 236 downregulated proteins (log2FC ≤ −1) and 137 upregulated proteins (log2FC > 1, *p* < 0.05).

We identified the sum of 24,160 peptides after conducting a TMT-based proteomic (Supplementary Table S1). According to the filter conditions, 373 differentially expressed proteins (DEPs) in NDSTB and DSTB samples were present in Figure 3, extracted from 5033 identified proteins (Supplementary Table S2, S3). Furthermore, we also demonstrated 137 upregulated proteins (log2FC ≤ −1) and 236 downregulated proteins (log2FC > 1, *p* < 0.05) between the NDSTB and DSTB groups.

**FIGURE 4:**
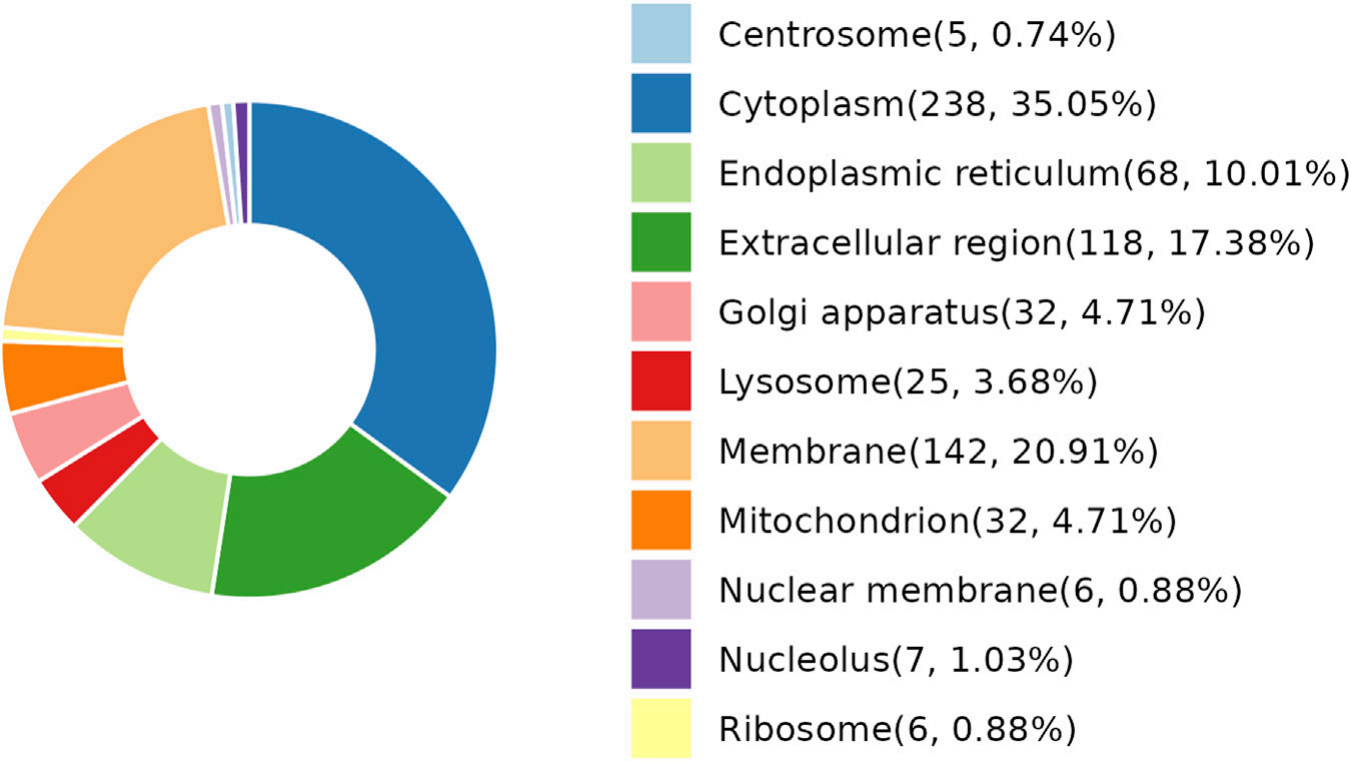
Subcellular localization analysis of DEPs. 25 DEPs were distributed in lysosomes, accounting for 3.68%. 238 DEPs were distributed in Cytoplasm, accounting for 35.05%.

As per the results demonstrated in this study, NDSTB and DSTB samples exhibited various differentially expressed proteins (DEPs). Analysis of the subcellular localization showed that DEPs were diffusely distributed throughout the cytoplasm, extracellular region, lysosomes, Golgi apparatus, centrosome, membrane, mitochondrion, nuclear membrane, nucleolus, ribosome, and endoplasmic reticulum (Figure 4).

### GO and kyoto encyclopedia of genes and genomes enrichment analysis for DEPs

**FIGURE 5:**
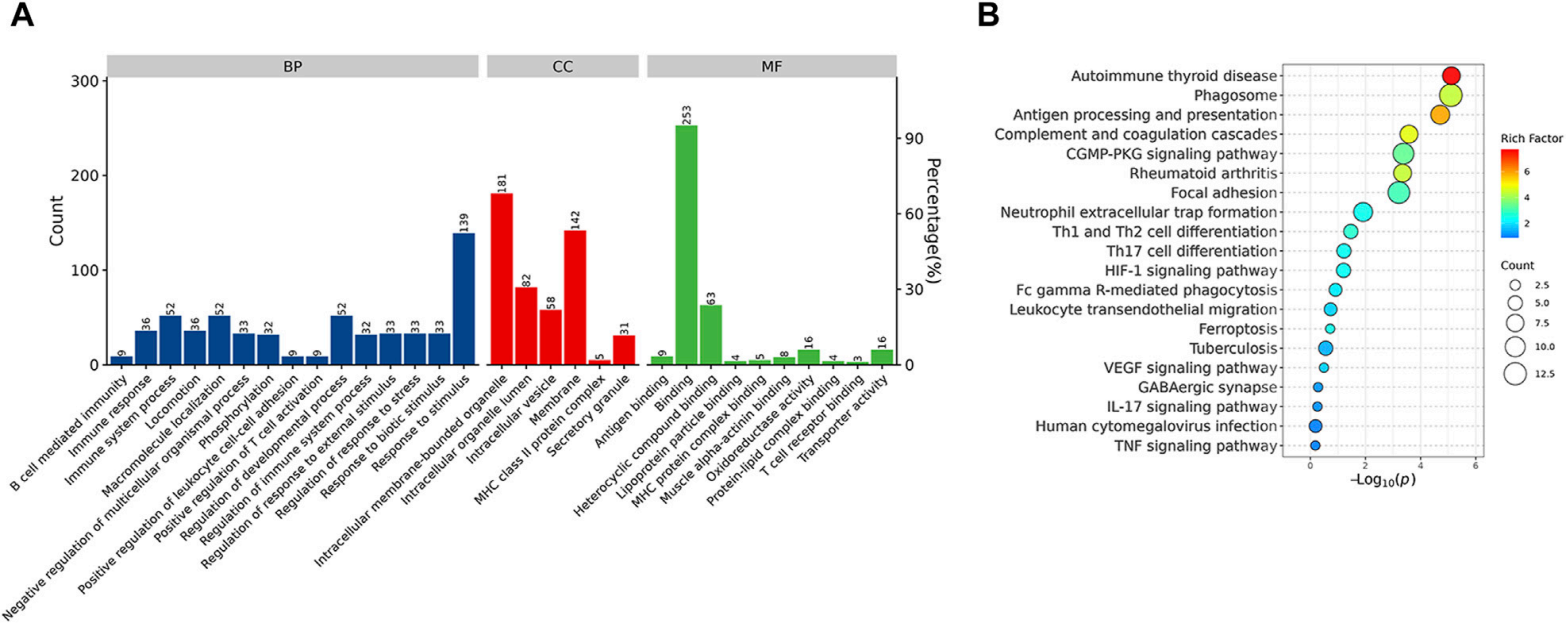
**(A)** BP, the cellular component category (CC), the molecular function category (MF) phantom enrich mentanalysis of DEPs. The left vertical coordinate is count (number of differential proteins annotated to the term), and the right vertical coordinate is percentage (number of differential proteins annotated to the term/total number of differential proteins with GO annotations). **(B)** KEGG enrichment pathway analysis of DEPs. The abscissa represents the negative logarithmic transformation of the Enrichment factor, and the ordinate represented the specific path. The column color indicates -log10 (*p*-value). Specific counts and *p*-values are presented on the right side of the column. KEGG, Kyoto Protocol Encyclopedia of Genes and Genomes; DEPs, differentially expressed proteins.

Functional enrichment analysis demonstrated that the DEPs were primarily involved in the immune system process and response to stimulus, which might be associated with the Rifampicin-resistant mechanism of spinal tuberculosis induced by the immune system process (Figure 5A). Notably, five proteins annotated with the term human leukocyte antigen, A Chain ((HLA-A), human leukocyte antigen, C Chain ((HLA-C), HLA Class II Histocompatibility Antigen, DRB1 Beta Chain (HLA-DRB1), metalloproteinase 9 (MMP9) and Phospholipase C-like 1 (PLCL1) were significantly changed compared with NDSTB (Supplementary Table S4). KEGG pathway analysis demonstrated that DEPs were mainly associated with “Antigen processing and presentation” and “Phagosome” (Figure 5B). These findings contribute novel perspectives to our understanding of the etiology and comorbidities associated with drug-resistant spinal tuberculosis.

### PPI analysis confirmed by Immunohistochemistry and Western blot analysis

**FIGURE 6:**
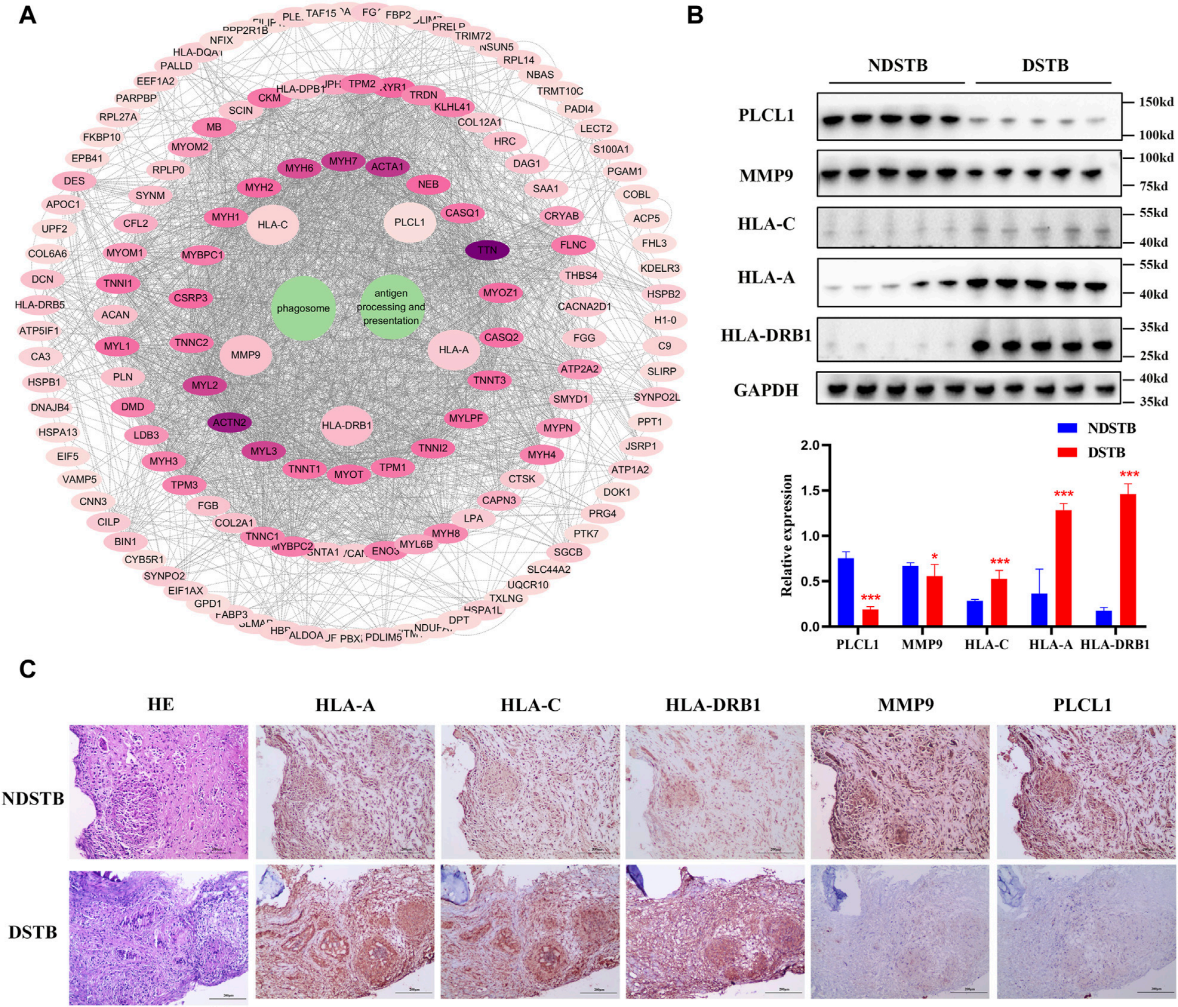
**(A)** “DEPs-drug-resistant spinal TB- “Antigen processing and presentation “pathway -“Phagosome” pathway -DEPs-Targets” network. Protein-protein interaction network using STRING. A PPI of all detected DEPs. The significantly enriched pathways (*p* < 0.05) and protein/protein coding gene and protein-protein interaction network associated with the pathways. The innermost circle is 5 of the key proteins of DEPs. The darker the color, the greater the degree value. The criteria for sub-setting was the more and denser the edges, the more central the protein was. The innermost five key proteins in the circle are most relevant to these two pathways. These proteins are particularly relevant to the mentioned pathways, with green highlighting their involvement in “Antigen processing and presentation” and “Phagosome.” **(B)** Relative expression of MMP9, PLCL1, HLA-A, HLA-C and HLA-DRB1 measured using the tissue protein of DSTB and NDSTB were tested by Western blot (*n* = 5). Statistically significant differences between groups were determined using a Student’s t-test or two-tailed one-way analysis of variance, followed by a Student-Newman-Keuls test; *p < 0.05. **(C)** IHC staining of tissue in the focal tissues of DSTB (*n* = 20) and NDSTB (*n* = 20) for MMP9, PLCL1, HLA-A, HLA-C and HLA-DRB1. scale bars = 200 µm.

Through KEGG signaling pathways analysis, it was revealed that HLA-DRB1, HLA-A, HLA-C, MMP9, and PLCL1 among the 25 DEPs were linked to “Antigen processing and presentation” and “Phagosome” pathways (as depicted in Figure 6A). To further exprole, results revealed that the expression of MMP9 and PLCL1 were remarkably decreased in the focal tissues of DSTB, compared with NDSTB. Contrarily, the HLA-A, HLA-C, and HLA-DRB1 were significantly increased in the focal tissues of DSTB, compared with NDSTB (Figures 6B,C), suggesting that five proteins (HLA-DRB1, HLA-A, HLA-C, MMP9 and PLCL1) may be involved in Rifampicin-resistant STB.

### Pharmaco-transcriptomic analysis

**FIGURE 7:**
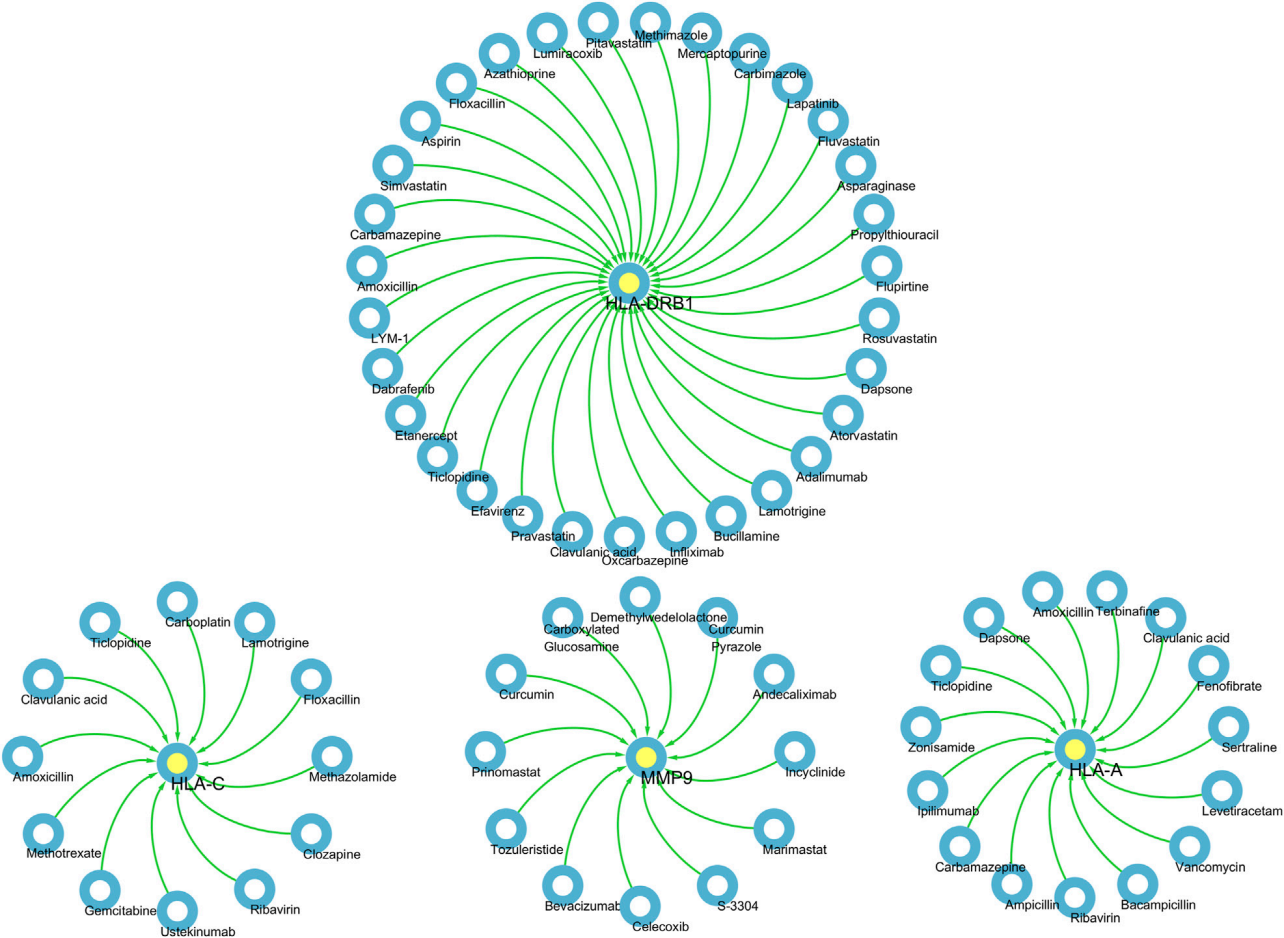
We performed pharmaco-transcriptomics analysis by the results of key proteins for the PPI network.pharmaco-transcriptomic analysis used the Integration of the Drug–Gene Interaction Database (DGIdb 4.0).

Based on the above results, HLA-A, HLA-C, HLA-DRB1, MMP9 and PLCL1 may be potential therapeutic targets for DSTB. Therefore, the results of our pharmaco-transcriptomic analysis can confirm that 13 drug compounds (e.g., Floxacillin, Clavulanic, and Amoxicillin) can regulate the expression of HLA-C. Similarly, there were 12 kinds of drug compounds found regulating MMP9, such as Celecoxib, Marimastat, and Andecaliximab. Moreover, 15 kinds of drug compounds (Terbinafine, Zonisamide, Ticlopidine, etc.) regulated the expression of HLA-A. In addition, Flupirtine, Fluvastatin, Methimazole, and other 18 drug compounds were confirmed to regulate the HLA-DRB1 (Figure 7). These experimental results will help us provide new insights into the treatment of DSTB.

## Discussion

In this study, TMT labeled Quantitative Proteomicswas employed to scrutinize the protein expression profiles of focal tissues in DSTB patients as compared to NDSTB. The identified signaling pathways, specifically “Antigen processing and presentation” and “Phagosome,” linked to DSTB, shed light on potential mechanisms underlying Rifampicin-resistance. Furthermore, five proteins (HLA-DRB1, HLA-A, HLA-C, MMP9 and PLCL1) were identified to be potential targets for DSTB.

It is worth noting that drug resistance of patients with spinal TB underlines at a higher rate under clinical use of four first-line drug combinations than theoretically predicted (Gao et al., 2016). Nahid et al. highlighted the choice and number of drugs significantly impact the speed of drug resistance spread, and exproled the mechanism of drug-resistant STB was extremely urgent (Nahid et al., 2019). Notably, our screening process identified five key proteins, HLA-DRB1, HLA-A, HLA-C, MMP9, and PLCL1, with distinct upregulation or downregulation in Rifampicin-resistant STB patients. Recent research has demonstrated that the HLA class II presentation of *Mycobacterium* bacterial antigens to CD4^+^ cells is one of the most crucial biological steps regarding the outcome of the infection (Magira et al., 2012), CD8 T lymphocytes recognize bacteria exposed at the membrane of infected cells. In addition, our study revealed the involvement of HLA-DRB1, HLA-A, HLA-C in the signaling pathways of antigen processing and presentation, suggesting their potential as targets by CD4^+^ for STB Rifampicin-resistance. MMP9 was reported closely involved in immune inflammation. Several cell types, including epithelial cells, macrophages, fibroblasts, and neutrophils, produce pro-MMP9 (Van den Steen et al., 2002). Therefore, MMP9-inhibiting monocytes can suppress the invasion of T lymphocytes into the walls of blood vessels, causing decreasing the density of an immune response that leads to vasculitis (Watanabe et al., 2018). These results indicate that HLA-DRB1, HLA-A, HLA-C, MMP9, and PLCL1 may play pivotal roles in the mechanism of Rifampicin-resistant spinal TB, potentially serving as diagnostic markers and promising therapeutic targets for DSTB.

Interestingly, HLA-DRB1, HLA-A and HLA-C were significantly increased in DSTB group, but MMP9 and PLCL1 were substantially decreased in DSTB group. The pharmaco-transcriptomic analysis identified various drugs (Floxacillin, Clavulanic, Ustekinumab and so on) capable of regulating the expression of these proteins. Ustekinumab, a human monoclonal antibody, was kown as a target inhibition of interleukin-12 and −23. Moreover, Morelli et al., 2022 demonstrated that interleukin-12/23 was related to HLA-C, since they preferentially present (auto) antigens, for example the metalloprotease (MMP) domain, to CD8 T cells. Our paper demonstrated that Ustekinumab could regulate the protein of HLA-C, which might be one of the mechanisms of Ustekinumab-induced upregulated risk of Rifampicin-resistant of spinal TB disease.

Moreover, our study faced certain limitations. Initially, the sample size was insufficient. Instead of conducting an analysis on a larger scale, we utilized only twenty pairs of 40 samples for the protein park test, which proved to be inadequate. Additionally, further validation is required through cell and animal experiments to bolster the findings of this study.

To sum up, the involvement of HLA-DRB1, HLA-A, HLA-C, MMP9, and PLCL1 in the mechanism of Rifampicin-resistant spinal tuberculosis is evident. These factors may serve as crucial components in both diagnosis and potential therapeutic targeting for Rifampicin-resistant spinal TB.

## Data Availability

The datasets presented in this study can be found in online repositories (https://www.scidb.cn/en/detail?dataSetId=64acfda7ef7546e694e45476c4766720&version=V1)/Supplementary Material.
